# Structural intervention at one bridge decreases the overall jumping suicide rate in Victoria, Australia

**DOI:** 10.1017/S2045796023000720

**Published:** 2023-09-18

**Authors:** J. Dwyer, M. J. Spittal, K. Scurrah, J. Pirkis, L. Bugeja, A. Clapperton

**Affiliations:** 1 Coroners Prevention Unit, Coroners Court of Victoria, Southbank, VIC, Australia; 2 Melbourne School of Population and Global Health, University of Melbourne, Melbourne, VIC, Australia; 3 Monash Nursing and Midwifery, Monash University, Clayton, VIC, Australia

**Keywords:** epidemiology, mental health, research design and methods, suicide

## Abstract

**Aims:**

There is clear evidence that installing safety barriers is effective in preventing jumping suicides from high-risk bridges with only moderate displacement to other nearby bridges. However, the impact of barriers on jumping suicides across broader geographical areas is not well understood. We examined patterns in jumping suicides across the state of Victoria, Australia, after a safety barrier was installed at the West Gate Bridge which, before the installation of the barrier, was the site of approximately 40% of Victoria’s jumping suicides.

**Methods:**

We used negative binomial regression analyses on Victorian data from 2000 to 2019 to compare rates of jumping suicides at the West Gate Bridge, other bridges and non-bridge jumping locations before, during and after the West Gate Bridge barrier installation. We conducted linear regression analyses to examine whether the distance travelled from the deceased’s usual residence to the location of their jumping suicide changed between the before, during and after barrier installation periods.

**Results:**

After installation of the barrier, there were no jumping suicides at the West Gate Bridge (rate ratio [RR] = 0.00, 95% credible intervals [95% Cr] = 0.00–0.0001) and there was strong evidence that the rate of jumping suicides at all locations declined by 65% (RR = 0.35, 95% Cr = 0.22–0.54). At other bridges, there was also evidence of a reduction (RR = 0.31, 95% Cr = 0.11–0.70), but there was no evidence of a change at non-bridge locations (RR = 0.74, 95% Cr = 0.39–1.30).

**Conclusion:**

After installation of the safety barrier at the West Gate Bridge, jumping suicide in Victoria decreased overall and at other bridges, and did not appear to change at non-bridge locations. Our findings show that when barriers are installed at a site responsible for a disproportionately high number of jumping suicides, they are not only highly effective at the site where the barriers are installed but can also have a prevention impact beyond the immediate locale at similar sites.

## Introduction

Installing a safety barrier at a bridge can prevent jumping suicides from occurring at that site, with minimal or no subsequent displacement of jumping suicides to other sites in the immediate locale, town or city where the bridge is located (Berman *et al.*, 2021; Cox *et al.*, 2013; Okolie *et al.*, 2020; Pirkis *et al.*, 2013). For example, a study of the effect of a barrier on the Gateway bridge in Brisbane, Australia, found no immediate displacement to another nearby bridge or other jumping locations (Law *et al.*, 2014), and a more recent study of the Ellington Bridge in Washington DC found no displacement to either an immediately adjacent bridge or to any other bridge in the city (Berman *et al.*, 2021). However, at present, there is little evidence to assist in understanding whether site displacement occurs across broader geographic areas, for example the region, state or country where the bridge is located.

Understanding the broader geography of site displacement is particularly pertinent when a safety barrier is installed at a bridge that has developed a reputation as a jumping suicide location, and where a high number of suicides occur. These bridges (referred to from here as ‘landmark’ jumping suicide locations, but also described in the literature as ‘hotspot’ or ‘iconic’ jumping suicide locations) can exert a significant attractive force beyond the immediate area where they are situated, with people sometimes travelling substantial distances and bypassing closer jumping locations (Lam *et al.*, 2017; Saeheim *et al.*, 2017; Seiden and Spence, 1984).

The West Gate Bridge in the south-eastern Australian state of Victoria (population 6.69 million people) was until recently a landmark suicide location. The West Gate Bridge is a large cable-stayed girder bridge spanning the Yarra River in Victoria’s capital city Melbourne. Its roadway reaches a maximum height of 58.5 m above water level. After construction was completed in 1978, it quickly became the most common location in Victoria for jumping suicide, accounting for nearly 40% of all such suicides in the state between 1990 and 2008; the next most common location accounted for only 1.6% of jumping suicides (personal communication, Coroners Court of Victoria, 2023). Work commenced on a safety barrier at the West Gate Bridge in March 2009 and was completed in April 2011 (Jamieson, 2012), and no further jumping suicides have occurred there to date (January 2023). The barrier is more than 3 m high, is cantilevered out from the edge of the bridge to prevent people from being able to put a ladder against it, has horizontal mesh which prevents people from wedging their fingers into the mesh, and it is topped by a smooth, rounded metal capping that offers no handholds.

In this study we examined whether the jumping suicide rate across the state of Victoria – including at both bridge and non-bridge locations – changed after the West Gate Bridge safety barrier was installed. As previous research has shown that individuals were willing to travel long distances to take their own lives at certain iconic or landmark bridges (Perron *et al.*, 2013), we also examined the distances that the deceased travelled from their usual residences to the West Gate Bridge and other jumping suicide locations, both before and after the safety barrier installation, to understand better the ‘attractive’ nature of landmark suicide locations.

## Method

### Study design

We conducted a case-series study of jumping suicides that occurred in Victoria, Australia between 1 January 2000 and 31 December 2019.

### Data sources

We sourced data for this study from the Victorian Suicide Register (VSR), which contains a core dataset of coded information for every suspected suicide in Victoria from 1 January 2000 to present, including the deceased’s sex, age, country of birth, usual residential address, suicide method and location of suicide. Suspected suicides are initially identified through daily surveillance of all deaths reported to the Coroners Court of Victoria. The contents of the VSR are continually revised and updated as coroners’ investigations progress and more information becomes known about the deaths (Sutherland *et al.*, 2018).

Population data for Victoria were sourced from the Australian Bureau of Statistics. This data series consists of quarterly estimates of the resident population and covered the entire study period. As the series only gave the population sizes for March, June, September and December quarters in each year, we applied the population size from the first month to the remaining two months in the quarter (e.g., March values were assigned to April and May).

### Case identification and coding

We extracted de-identified data from the VSR for all suicides and for every jumping suicide that occurred between 1 January 2000 and 31 December 2019. Jumping suicides included deaths that occurred in circumstances where (a) the cause of death was jumping or falling from a high place; (b) the available evidence indicated on the balance of probabilities that the deceased intentionally jumped or fell; and (c) the available evidence indicated on the balance of probabilities that the deceased understood and intended that death would be the likely result of jumping or falling (ICD-10 code X80). The data for all suicides were aggregated to the monthly level. The dataset on jumping suicides were at the unit record level and included information on the deceased’s sex (female, male), age group (<25, 25–34, 35–44, 45–54, 55–64, ≥65 years), date of death report, latitude–longitude coordinates of usual residence and latitude–longitude coordinates of suicide location.

We classified each jumping suicide location into one of three groups: the West Gate Bridge, other bridges (including road, rail and pedestrian bridges) and non-bridge locations (including residential and office buildings, cliffs, lookout towers and hotels). For each suicide, we used the haversine formula (an accurate way of computing distances between two points on the surface of a sphere using the latitude and longitude of the two points) to calculate the straight-line distance (in kilometres) from the deceased’s usual residence to the jumping location using the latitude–longitude coordinates of each.

### Exposure variable

The before, during and after periods of barrier installation were defined as follows. Barrier installation began in March 2009. All jumping suicides before this month were classified as occurring in the before period (total = 110 months). Barrier installation was completed in April 2011 (total months = 25 months). All suicides between April 2009 and March 2011 were classified as occurring in the during period, and all suicides from April 2011 onwards were classified as occurring in the after period (total = 105 months).

### Statistical analyses

Jumping suicides: To examine whether there were changes in the rate of jumping suicides (the outcome variable) between the three time periods, we aggregated the jumping suicide data to the monthly level overall and by the three locations. We merged this with the monthly data of all suicides and the monthly population data. We then fit a negative binomial regression to this data with indicator variables for exposure periods (before, during and after with the before period used as the reference), a linear time trend variable to control for the potential confounding effects of time, and the population size in each month as an offset term. Models were stratified by location (all jumping suicides, the three jumping locations and all suicides). Because there were zero counts in the number of monthly suicides post-installation at one location, all four models were fit using Bayesian estimation techniques with non-informative priors for all parameters, 5,000 burn-in iterations and 20,000 MCMC simulations. We report the mean rate ratio [RR] from these simulations and their 95% credible intervals (95% Cr).

Distance travelled: To examine whether there were differences in the distance travelled to a jumping suicide location during the three time periods, we first report the median distance travelled for each location by period. We then fit linear regression models to the distance travelled variable. Only jumping suicides that occurred away from the usual residence, within the state of Victoria and where the deceased had a known address were included in this analysis. The outcome variable, distance travelled in kilometres, was transformed using the natural logarithm to accommodate skew. Models were stratified by location and each model included indicator variables for exposure period (as above) and covariates for age (<25, 25–34, 35–44, 45–54, 55–64, ≥65 years with <25 group used as the reference) and sex (female, male with female used as the reference). Model coefficients were back-transformed for interpretation and represent the percentage change in the distance travelled (with 95% confidence interval, 95% CI).

### Ethical review

The study was reviewed and approved by the University of Melbourne’s Human Research Ethics Committee (Reference Number: 2021-22015-21133-3). Access to the VSR data for the purposes of this study was granted by the Victorian State Coroner.

## Results

**Figure 1.**
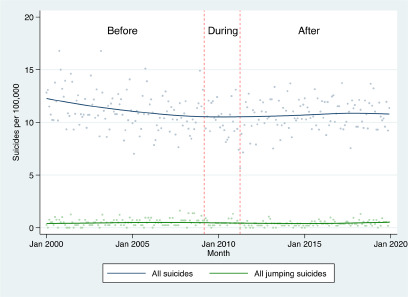
Trend in all suicides and jumping suicides, January 2000 to December 2019.

*Note:* Trend line calculated using a Lowess smoother.

There was a total of 11,903 suicides recorded between 1 January 2000 and 31 December 2019 in the VSR (Fig. 1). Of these suicides, 491 were jumping suicides (4.1%) and these are the focus of the following analyses. A total of 104 jumping suicides were from the West Gate Bridge (21.2%), 118 from other bridges (24.0%) and 269 from non-bridge locations (54.8%) (Table 1). Men were overrepresented (73.7% of the total with similar proportions at each location), and the most common age group was 25–34 years (27.7% of the total). Before installation of the barrier, the most common location for jumping suicide was the West Gate Bridge (95 suicides, 41.1% of jumping suicides) followed by non-bridge locations (93 suicides, 40.3% of jumping suicides). After installation of the barrier at the West Gate Bridge, no suicides occurred at this location and 143 suicides occurred at non-bridge locations.

**Table 1.** Number of jumping suicides in Victoria by location of suicide (*n* = 491)

	All jumping locations (*n* = 491)		West Gate Bridge (*n* = 104)		Other bridges (*n* = 118)		Non-bridge locations (*n* = 269)	
	*N*	%	*N*	%	*N*	%	*N*	%
**Sex**								
Female	129	26.3	24	23.1	25	21.2	80	29.7
Male	362	73.7	80	76.9	93	78.8	189	70.3
**Age group**								
Under 25	101	20.6	21	20.2	23	19.5	57	21.2
25–34	136	27.7	34	32.7	31	26.3	71	26.4
35–44	107	21.8	29	27.9	23	19.5	55	20.4
45–54	69	14.1	14	13.5	23	19.5	32	11.9
55–64	50	10.2	6	5.8	12	10.2	32	11.9
65 and over	28	5.7	0	0.0	6	5.1	22	8.2
**Period**								
Before	231	47.0	95	91.3	43	36.4	93	34.6
During	55	11.2	9	8.7	13	11.0	33	12.3
After	205	41.8	0	0.0	62	52.5	143	53.2

Note: Before period = January 2000 to February 2009 (110 months), During period = March 2009 to March 2011 (25 months), After period = April 2011 to December 2019 (105 months).

### Jumping suicide before, during and after barrier installation

Jumping suicide accounted for 4.6% of all suicides before the installation of the West Gate Bridge barrier, 4.7% during installation and 3.6% after installation. In comparison to the pre-installation period and after controlling for time trends, there was evidence that the jumping suicide rate at all locations was 34% lower than expected during the period the barrier was installed on the West Gate Bridge (RR = 0.66, 95% Cr = 0.46–0.92) (Table 2). There was also evidence that the rate at all locations was 65% lower than expected in the post-installation period (RR = 0.35, 95% Cr = 0.22–0.54).

**Table 2.** Rate ratios (RR) comparing the number of jumping suicides in the period before the installation of a barrier on the West Gate Bridge to the periods during and after installation

Location	Period	Number of suicides	Crude rate per 100,000 person-years	Adjusted rate ratio (95% credible interval)
All jumping locations	Before	231	0.51	Ref.
	During	55	0.49	0.66 (0.46–0.92)
	After	205	0.39	0.35 (0.22–0.54)
West Gate Bridge	Before	95	0.21	Ref.
	During	9	0.08	0.31 (0.12–0.62)
	After	0	0.00	0.00 (0.00–0.0001)
Other bridges	Before	43	0.09	Ref.
	During	13	0.11	0.63 (0.27–1.18)
	After	62	0.12	0.31 (0.11–0.70)
Non-bridge locations	Before	93	0.20	Ref.
	During	33	0.29	1.08 (0.65–1.67)
	After	143	0.27	0.74 (0.39–1.30)
All suicides	Before	5062	11.2	Ref.
	During	1160	10.3	0.94 (0.87–1.02)
	After	5681	10.7	1.01 (0.93–1.11)

Note: Before period = January 2000 to February 2009 (110 months), During period = March 2009 to March 2011 (25 months), After period = April 2011 to December 2019 (105 months). All models fit using negative binomial regression with Bayesian estimation and adjusted for time and population size.

When locations were considered separately, there was evidence of a lower than expected suicide rate at the West Gate Bridge. In comparison to the pre-installation period, the expected rate declined 69% during installation (RR = 0.31, 95% Cr = 0.12–0.62) and 100% post installation (RR = 0.00, 95% Cr = 0.00–0.0001). At other bridges, there was no evidence of a change in the expected suicide rate during the installation of a barrier at the West Gate Bridge (RR = 0.63, 95% Cr = 0.27–1.18) but evidence of a lower than expected rate post installation (RR = 0.31, 95% Cr = 0.11–0.70). There was no evidence of a change in the suicide rate at non-bridge locations (RR = 1.08, 95% Cr = 0.65–1.67 during installation and RR = 0.74, 95% Cr = 0.39–1.30 post installation).

**Figure 2.**
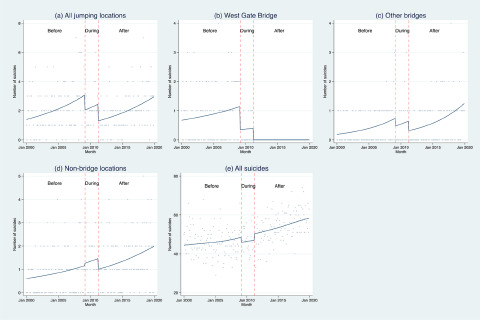
Observed and fitted number of suicides, January 2000 to December 2019.

Finally, when the analysis was repeated for all suicides, there was no evidence of a change in the expected suicides rate during or after installation of the barrier (RR = 0.94, 95% Cr = 0.87–1.02 during installation and RR = 1.01, 95% Cr = 0.93–1.11 post installation). Observed and fitted values for all these results are plotted in Fig. 2.

### Distance travelled

Of the 491 jumping suicides, there was valid data on distance travelled from usual residence to the jumping suicide location for 388 individuals (79% of all jumping suicides). Over the whole study period, the median distance travelled to the West Gate Bridge was 14.9 km, the median distance travelled to other bridges was 3.1 km and the median distance travelled to non-bridge locations was 8.0 km.

For all locations, the median distance travelled before installation of the barrier at the West Gate Bridge was 11.0 km, during installation it was 7.6 km and post installation it was 7.7 km (Table 3). There was no evidence that these distances varied over the three periods (i.e., the CIs for the percentage change in the distance travelled during and post installation both included the null value of 0 which indicates no change). This same finding was observed when the analyses were stratified by location, including at the West Gate Bridge.

**Table 3.** Distance travelled from usual residence to jumping locations before, during and after the installation of a barrier on the West Gate Bridge, *n* = 388 suicides

Location	Period	Number of suicides	Median distance travelled (km)	Percentage change in distance travelled (95% confidence Interval)
All jumping locations	Before	196	11.0	Ref.
	During	48	7.6	−22.6% (−60.2 to 15.0%)
	After	144	7.7	−22.4% (−48.1 to 3.3%)
West Gate Bridge	Before	95	14.8	Ref.
	During	9	23.5	30.2% (−37.3 to 97.7%)
	After	0	–	–
Other bridges	Before	43	2.5	Ref.
	During	13	2.5	−21.4% (−94.1 to 51.3%)
	After	60	3.9	44.6% (−41.0 to 130.1%)
Non-bridge locations	Before	58	7.1	Ref.
	During	26	6.6	−5.4% (−81.2 to 70.3%)
	After	84	9.8	32.8% (−42.6 to 108.1%)

Note: Before period = January 2000 to February 2009 (110 months), During period = March 2009 to March 2011 (25 months), After period = April 2011 to December 2019 (105 months). Estimates for percentage change in distance travelled fit using linear regression with a log transformation for the outcome variable and adjusted for age and sex.

## Discussion

We examined the effectiveness of the installation of a barrier on Victoria’s West Gate Bridge as a jumping suicide prevention measure at that site. In addition, we examined whether there was a change in the rate of jumping suicides at all other bridges and at non-bridge locations across the state of Victoria after the installation of the barrier on the West Gate Bridge. We found very strong evidence of a reduction in jumping suicides at the West Gate Bridge (100% reduction), and there was no evidence of site displacement to other bridges or to non-bridge locations. Rather than site displacement, we found some evidence of a decrease in jumping suicides at other bridges (69% reduction), and no change in the rate of jumping suicides at non-bridge locations. Putting all these results together, we found an overall 65% reduction in jumping suicides across the state of Victoria after the barrier installation on the West Gate Bridge.

There have been no suicides from the West Gate Bridge since the installation of the barrier (now nearly 12 years ago), which contrasts with some other bridges where suicides continued to occur post installation of a barrier (Bennewith *et al.*, 2007, 2011; Hemmer *et al.*, 2017; Law *et al.*, 2014; Perron *et al.*, 2013). This suggests the specific characteristics of the West Gate Bridge barrier may be highly effective. The West Gate Bridge barrier complies with all the characteristics that were found in a recent study (Hemmer *et al.*, 2017) to be associated with a complete elimination of suicide, namely that a barrier is very high (at least 2.3 m), secures the jump site across the entire length and prevents climbing around the bridgeheads (Hemmer *et al.*, 2017).

Our findings are largely consistent with those of other published studies that have showed that restricting access to the means of suicide at a bridge site can decrease the incidence of suicide at that site (Beautrais, 2001; Beautrais *et al.*, 2009; Bennewith *et al.*, 2007, 2011; Berman *et al.*, 2021; Law *et al.*, 2014; Lester, 1993; Pelletier, 2007; Perron *et al.*, 2013) and that the installation does not lead to displacement of that same method at another nearby bridge or bridges (Berman *et al.*, 2021; Law *et al.*, 2014; Perron *et al.*, 2013). However, we extended on previous studies by examining not only the effect of the installation of a barrier on one bridge and surrounding bridges but also examining the effect on all jumping suicides at all locations across an entire state (population of approximately 6.69 million people).

A finding that is unique to our study is the identified reduction in jumping suicide at other bridges across a large geographical area (the entire state of Victoria) rather than simply at the immediate locale where the barrier was installed. This is a promising, if unexpected, finding. It should be acknowledged that the potential confounding effect of structural intervention at other bridges could have influenced this result. For example, following several coroners’ recommendations (e.g., Jamieson, 2015), in 2018 a safety barrier was erected along the EJ Whitten Bridge in Melbourne’s west, which had become the most common bridge for jumping suicides in Victoria following the West Gate Bridge safety barrier installation. Although notable, we do not believe this is what is driving the decrease because our study period was 2000 to 2019 and therefore only included one full year post installation of this barrier on another bridge. Perhaps a clue to why we found this lies in the concept of ‘cognitive availability’ of suicide methods. This is the idea that how accessible something is in one’s mind can play a role in suicide method choice (Florentine and Crane, 2010). For many decades, the West Gate Bridge was synonymous with jumping suicide in Victoria. Maybe installing a safety barrier not only addressed suicide at the West Gate Bridge, but also potentially reduced discussion of and reference to suicide by jumping across the state (i.e., reduced the cognitive availability of jumping from bridges as a suicide method) thereby resulting in a reduction in jumping suicide from all bridges across the state.

One argument often put forward against structural intervention at a site where jumping suicides occur, is that if a barrier prevents individuals from jumping at one site, then they will jump at another site (site displacement) (Ironside, 2012). A second argument is that people will use a different suicide method if their ‘preferred’ method is unavailable (method substitution) (Ironside, 2012). The results of our study allow us to refute the first argument, given that we found no increase occurred at other jumping sites after barrier installation at the West Gate Bridge, but we did not examine method substitution in our study. In the case of suicide by jumping, existing evidence suggests displacement to other jumping locations is probably more likely than a change in method (Okolie *et al.*, 2020; Perron *et al.*, 2013); therefore, given we found no evidence of site displacement, it seems promising that method substitution might also not be occurring.

People travelling substantial distances to jump at specific bridges has been documented in previous studies (Glasgow, 2011; Law *et al.*, 2014; Perron *et al.*, 2013; Seiden and Spence, 1984), so it is unsurprising that we found that in the period before barrier installation individuals travelled further to the West Gate Bridge than they did to other bridges. We found little evidence of a change in the average distance travelled to other bridges and to non-bridge locations after the installation of the barrier on the West Gate Bridge. These results are promising as they are consistent with an interpretation that people who would have travelled a significant distance to jump from the West Gate Bridge are not now travelling a significant distance to jump from other locations. Or, to put it another way, the ‘attractive’ force of the West Gate Bridge does not appear to have transferred to other locations.

### Strengths and limitations

The main strength of our study is that we were able to examine whether site displacement occurs at a broad population level by including data for all jumping suicides across the state of Victoria. Another strength of the study is that we used manually assigned, geocoded suicide location data rather than relying on data based on auto-geocoding processes which are known to be inaccurate for non-residential addresses (Torok *et al.*, 2021). The availability of lengthy pre-and post-intervention periods of data was a strength of the study, especially given that short-term findings from studies regarding reducing access to means can be misleading (Sinyor *et al.*, 2017).

Despite these strengths, our study has limitations. Although we showed there was no evidence for site displacement after the installation of the barrier on the West Gate Bridge, as mentioned, we did not examine the potential substitution effects whereby individuals may use another suicide method after the installation of the barrier. Additionally, we did not consider whether the installation of the barrier on the West Gate Bridge was cost effective, however; recent research suggests this is likely to be the case (Bandara *et al.*, 2022). In addition, our models assumed that the time trend was the same between periods, but it is possible that the slope of the line for time differed between periods. The low number of jumping suicides in each month meant it was not possible to test this hypothesis. Finally, in our analysis of distance travelled, we calculated distance using the straight-line distance from the deceased’s usual residence to the jumping location. We acknowledge that this could be misleading in some cases (e.g., due to the presence of geographical features such as mountains or rivers).

### Implications and conclusion

Consistent with strong existing evidence that restricting access to the means of jumping at sites through installing barriers is an effective strategy to prevent suicide at these sites (Berman *et al.*, 2021; Cox *et al.*, 2013; Okolie *et al.*, 2020; Pirkis *et al.*, 2013, 2015), our study showed jumping suicides stopped at the West Gate Bridge after the installation of a barrier. Further, we showed there was no evidence of an increase in suicides at other jumping locations (i.e., bridges or non-bridge locations) across the state of Victoria following the introduction of the barrier. Clearly, the installation of the barrier on the West Gate Bridge has been effective and has saved lives. Therefore, we believe this research provides evidence that barriers should be retrospectively installed at known jumping locations, and as part of the design and building of all new bridges, barriers should be considered from the beginning as their inclusion should prevent bridges becoming sites that attract people to jump.

The West Gate Bridge safety barrier installation was not a stand-alone project, but occurred together with widening and strengthening works, the total cost of which reached AUD$371 million (Zhang, 2018). In this respect, the West Gate Bridge approach might hold lessons for those working to address other landmark suicide locations around the world. Where the cost of structural interventions is considered to be prohibitively high, they might be more likely to be funded and implemented if they are bundled with infrastructure improvements. Further to this point, the West Gate Bridge safety barrier installation was followed by a decline in jumping suicides across the entire state of Victoria; this suggests that modelling for the likely impact of structural interventions at other landmark locations might need to include the possibility of a suicide prevention effect well outside the immediate environs of the landmark location itself. Noting the desirability of primary prevention, it is crucial that large infrastructure projects such as high bridges incorporate suicide prevention principles into their design from the outset (Clapperton *et al.*, 2022).

## Data Availability

Due to the sensitivity of the suicide data, the data on which this manuscript are based are not publicly available. Data can be requested from the Coroners Court of Victoria.
